# A Small-Sample Fault Diagnosis Method for High-Voltage Circuit Breaker Spring Mechanisms Based on Multi-Source Feature Fusion and Stacking Ensemble Learning

**DOI:** 10.3390/s26051485

**Published:** 2026-02-26

**Authors:** Xining Li, Hanyan Xiao, Ke Zhao, Lei Sun, Tianxin Zhuang, Haoyan Zhang, Hongwei Mei

**Affiliations:** 1 State Grid Jiangsu Electric Power Research Institute, Nanjing 211103, China; 1320211433@139.com (X.L.); xiaohy1@js.sgcc.com.cn (H.X.); zhaok@js.sgcc.com.cn (K.Z.); sunl12@js.sgcc.com.cn (L.S.); zhuangtx@js.sgcc.com.cn (T.Z.); 2 Shenzhen International Graduate School, Tsinghua University, Shenzhen 518055, China; haoyan-z24@mails.tsinghua.edu.cn

**Keywords:** high-voltage circuit breaker, fault diagnosis, small sample, multi-source feature fusion, stacking ensemble learning, MEMS sensor

## Abstract

To address the practical engineering challenges of limited fault samples for high-voltage circuit breaker spring operating mechanisms and the inability of single features to fully reflect equipment status, this paper proposes a small-sample fault diagnosis method based on multi-source feature fusion and Stacking ensemble learning. First, a multi-source sensing system containing MEMS (Micro-Electro-Mechanical System) pressure and travel, coil, and motor current was constructed to achieve comprehensive monitoring of the mechanical and electrical states of a 220 kV circuit breaker; in particular, the introduction of non-invasive MEMS sensors effectively solves the difficulty of capturing static spring fatigue characteristics inherent in traditional methods. Second, a high-dimensional feature space was constructed using Savitzky–Golay filtering and physical feature extraction techniques. To address the characteristics of small-sample data distribution, a two-layer Stacking ensemble learning model based on 5-fold cross-validation was designed. This model utilizes the SVM (Support Vector Machine), RF (Random Forest), and KNN (K-Nearest Neighbors) as base classifiers and Logistic Regression as the meta-learner, achieving an adaptive fusion of the advantages of heterogeneous algorithms. True-type experimental results show that the average diagnostic accuracy of this method under normal conditions and four typical fault conditions reaches 96.1%, which is superior to single base models (the RF was 94.2%). Feature importance analysis further confirms that closing and opening pressures are the most critical features for distinguishing mechanical faults. This study provides effective theoretical basis and technical support for condition-based maintenance of high-voltage circuit breakers under small-sample conditions.

## 1. Introduction

**Figure 1:**
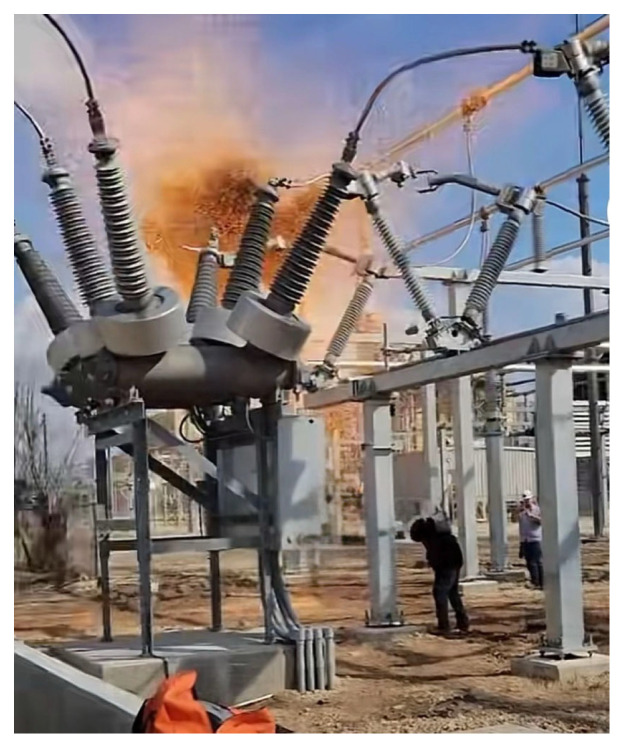
GIS spring circuit breaker explosion accident.

With the advancement of new power system construction and the increasing complexity of ultra-high-voltage backbone networks, the safe and stable operation of the power grid imposes unprecedentedly strict requirements on the reliability of key primary equipment. As a defensive gateway possessing both control and protection functions in the power grid, the operating performance of high-voltage circuit breakers directly determines the timeliness of fault clearing and the stability of the power supply system [1,2,3]. However, during long-term service, under the multiple influences of environmental stress, mechanical wear, and material aging, the health status of circuit breakers inevitably shows a deterioration trend. Once a failure to operate, maloperation, or jamming fault occurs, it can lead to leapfrog tripping, expanding the scope of power outages or even causing grid oscillation and system collapse, resulting in huge economic losses and social impact [4]. Figure 1 shows a transformer explosion accident in a certain area.

Among various high-voltage circuit breaker faults, mechanical faults caused by the operating mechanism have long occupied a dominant position. According to statistics from CIGRE and power companies in various regions, mechanical faults account for 40–50% of total circuit breaker faults year-round, far exceeding insulation faults and current-carrying faults. Among them, spring operating mechanisms are widely used in voltage levels of 126 kV and below due to their compact structure, high output power, and low maintenance workload [5]. However, this type of mechanism contains hundreds of precision components such as energy storage motors, opening/closing springs, cam transmission chains, and buffers and operates under special conditions of “long-term static standby and instantaneous action”. This alternating action of long-term stasis and instantaneous high-stress release can easily lead to spring stress relaxation, grease drying and hardening, and changes in component fit clearances. Therefore, breaking through the bottleneck of state perception for old spring mechanisms and realizing the transformation of operation and maintenance modes from “post-event repair” to “pre-event warning” has become a key technical problem to be solved urgently in the power industry [6,7,8].

**Figure 2:**
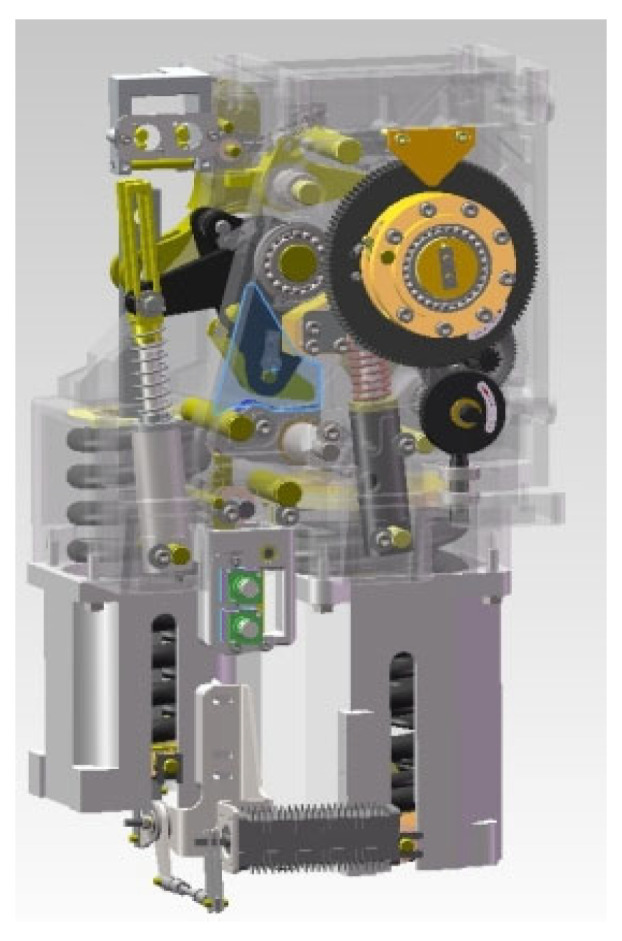
Schematic diagram of the spring circuit breaker operating mechanism.

However, the number of operations of a spring circuit breaker within its life cycle is limited. Due to long-term exposure to harsh outdoor environments, the performance of the circuit breaker may gradually decline with increasing service years. Specifically, in outdoor harsh working conditions such as coastal areas, areas with high humidity and heat, or those with heavy industrial pollution, circuit breakers are constantly exposed to high humidity and salt spray erosion. The schematic diagram of the operating mechanism of the spring-type circuit breaker is shown in Figure 2. The precise metal components inside the operating mechanism (such as transmission rods, rotating pin shafts, and contact surfaces of locks) are highly prone to atmospheric corrosion and electrochemical corrosion. As the service time progresses, the accumulation of corrosion products not only damages the smoothness of the component surfaces and the lubricating oil film but also significantly increases the friction resistance of the mechanical transmission chain. In severe cases, it may even cause rusting and solidification of key moving parts or hydrogen embrittlement fractures of springs. This material degradation induced by environmental corrosion is often more concealed than simple mechanical wear and tear. It is an important latent cause that can trigger jamming, failure to operate, or an abnormal reduction in action speed of the mechanism [9]. Under the superposition of complex environmental factors and mechanical loss, high-voltage circuit breakers have gradually become a weak link in the power system. High-voltage circuit breakers may experience faults such as failure to operate or maloperation, which not only affect the normal operation of equipment but also seriously threaten the safety and reliability of the power system [10,11].

For a long time, the maintenance of high-voltage circuit breakers in the power industry has mainly followed the periodic planned maintenance mode [12]. This mode relies on the “Preventive Test Code for Electric Power Equipment,” conducting power-off disassembly and overhaul according to fixed time cycles (e.g., 3–6 years). Maintenance content mainly relies on manual experience, judging equipment status through manual energy storage tests and measuring opening/closing times and speeds with mechanical characteristic testers. However, with the surge in grid equipment inventory, this traditional maintenance mode has gradually exposed serious limitations. (1) For equipment in good condition, forced disassembly not only causes a huge waste of manpower and material resources, but frequent disassembly and assembly may also destroy original factory assembly precision, leading to “repair-induced damage”; for equipment with familial defects or hidden dangers, fixed maintenance cycles often lag behind fault occurrence times, leading to operation with defects. (2) Spring operating mechanisms possess hundreds of precision-fitted components; traditional maintenance often requires invasive disassembly. This blind disassembly method cannot reproduce the true stress state of the equipment under operating conditions and can easily introduce new minute assembly errors, reducing the long-term reliability of the equipment [13].

In view of this, the transition from periodic maintenance to condition-based maintenance (CBM) based on real-time equipment health status has become a consensus in the global power industry [14]. The core premise of realizing condition-based maintenance lies in the precise perception and real-time diagnosis of equipment operating status.

To support condition-based maintenance, scholars at home and abroad have developed various non-invasive monitoring technologies, among which opening/closing coil current and mechanical vibration signals are most widely used. Coil current can effectively invert the core motion state and the integrity of the control loop by extracting the feature points of the current waveform (such as start time and peak time). However, coil current is essentially an electrical representation of the electromagnetic conversion process; its sensitivity to load changes at the end of the mechanical transmission chain (such as the main shaft and contacts) is low, making it difficult to accurately identify jamming defects deep within the mechanism [15,16,17,18]. Using acceleration sensors to capture shock signals at the moment of mechanism action allows rich mechanical status information to be collected. But at engineering sites, vibration signals are easily affected by strong electromagnetic interference in substations and resonance of the circuit breaker body, and non-invasive installation methods lead to complex signal transmission paths, severe high-frequency attenuation, and difficulty in guaranteeing signal-to-noise ratios [19,20,21]. Whether it is current or vibration, they are dynamic monitoring methods, meaning that data can only be obtained when the circuit breaker operates. However, spring fatigue and stress relaxation are slow static processes. This results in operation and maintenance personnel being unable to accurately know whether energy storage is sufficient when the circuit breaker is static, often only discovering spring failure when a failure to operate occurs [22].

After acquiring monitoring data, extracting fault features from the data is another major difficulty. With the penetration of artificial intelligence technology, data-driven methods have gradually replaced traditional threshold judgment methods. In recent years, models such as Convolutional Neural Networks (CNNs), Long Short-Term Memory (LSTM) networks, and Deep Belief Networks (DBNs) have been widely used in circuit breaker fault classification, demonstrating strong self-extraction capabilities for features [23,24,25,26]. However, the success of deep learning relies heavily on massive labeled data. In actual grid operation, the number of circuit breaker operations is extremely limited (some equipment operates only 1–2 times a year), and fault samples present a typical long-tail distribution—normal samples are readily available, while real fatigue, fracture, and other fault samples are extremely scarce [27,28,29]. In the context of actual power grid engineering, acquiring massive fault datasets is practically impossible. Therefore, the term “small sample” or “limited sample” in this study specifically refers to the restricted sample size relative to the demands of data-hungry deep learning models. While a dataset of hundreds of samples is not “extremely small” for traditional machine learning algorithms, directly applying deep neural networks designed for big data to such a restricted dataset can easily lead to model overfitting, poor generalization ability, and high false alarm rates on site. In contrast, ensemble learning completes learning tasks by constructing and combining multiple base learners, possessing natural advantages in handling small-sample, high-dimensional, and noisy data. However, research on the fusion diagnosis of multi-source heterogeneous data on circuit breakers is still in its infancy.

The organization of this paper is as follows: Section 2 details the experimental platform and the multi-source sensing system, including the 220 kV spring circuit breaker and its equipped MEMS pressure sensors, non-contact travel sensors, and current sensors for opening/closing and energy storage motors. It also describes the method of acquiring experimental data by manually simulating typical faults such as spring fatigue, coil voltage deviations, and motor aging. Section 3 focuses on data preprocessing and feature engineering, including the use of Savitzky–Golay filtering to remove high-frequency noise and the extraction of multi-dimensional physical features such as static pressure, peak current, and travel speed. Section 4 constructs a two-layer Stacking ensemble learning model based on 5-fold cross-validation, elaborating on the rationale for selecting heterogeneous base classifiers (SVM, RF, and KNN) and the adaptive fusion mechanism of the meta-learner. The effectiveness of this method in small-sample fault diagnosis is verified through confusion matrices and feature importance analysis. Finally, Section 5 summarizes the research conclusions and provides an outlook for future work.

## 2. Experimental Platform and Fault Simulation

**Figure 3:**
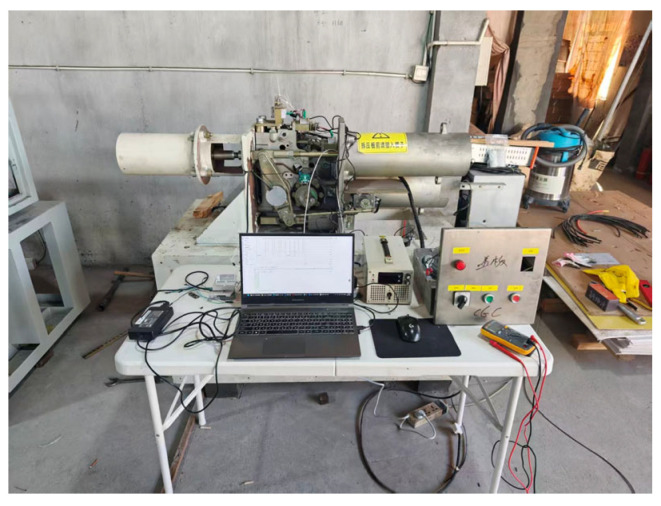
The 220 kV spring operating mechanism.

The experiments in this paper are based on a 220 kV spring circuit breaker mechanism as shown in Figure 3, in which a multi-sensor system is installed, including closing pressure sensors, opening pressure sensors, opening/closing current sensors, energy storage motor current sensors, and opening/closing travel sensors. The experimental platform mainly consists of the 220 kV circuit breaker body, a spring operating mechanism box, a multi-source sensor monitoring array, a high-frequency data acquisition unit, and an industrial control computer terminal. This sensor system can monitor the state changes in the operating mechanism during opening and closing actions in real time, providing data support for fault diagnosis.

### 2.1. Sensors and Installation

**Figure 4:**
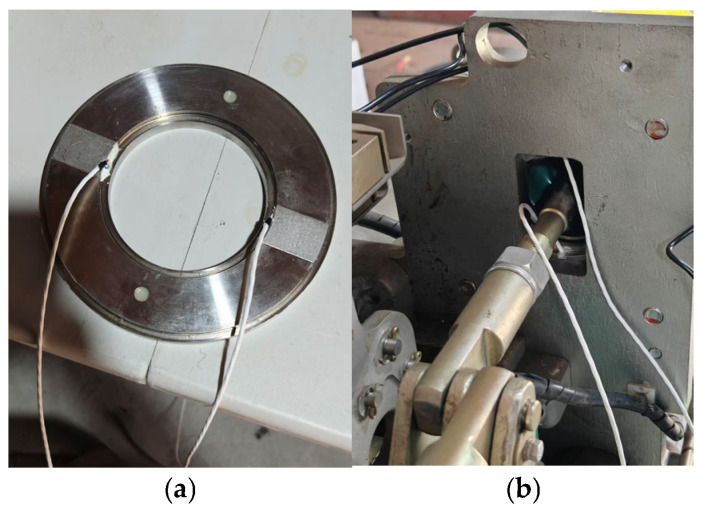
MEMS pressure sensor: (**a**) physical sensor and (**b**) sensor assembly diagram.

As shown in Figure 4, this study selected a ring-type MEMS pressure sensor based on the principle of hydraulic conduction. Its core mechanism lies in using hydraulic oil as an intermediate medium to convert the axial mechanical force generated by spring compression into fluid pressure within a hydraulic chamber, thereby achieving long-term precise perception of static elastic force. In terms of engineering deployment, the sensor adopts an embedded installation scheme, where it was placed between the spring and the end pressure plate. Its unique coaxial ring configuration ensures non-destructive monitoring without interfering with the original spring assembly structure. Sensors of different sizes were developed for the opening and closing spring sleeves respectively to meet the measurement needs of opening pressure and closing pressure.

**Figure 5:**
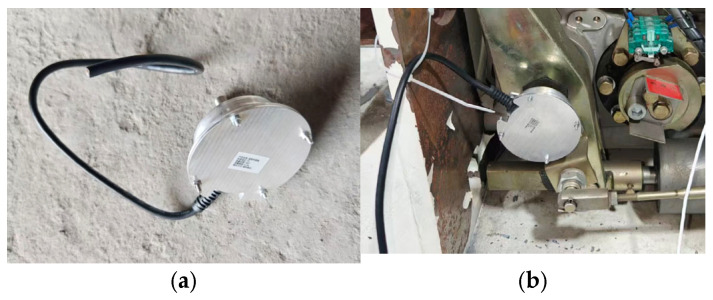
Opening/closing travel sensor: (**a**) physical sensor and (**b**) sensor assembly diagram.

Regarding the selection of opening/closing travel sensors, Figure 5 shows the non-contact opening/closing speed measurement device adopted by this system. The device applies a split design of a sensor and a code disk, where the code disk is installed in the reserved hole of the circuit breaker, and the encoder is flexibly mounted via a customized bracket. This structural design facilitates on-site assembly alignment while effectively eliminating vibration interference through flexible connection, significantly improving the shock resistance and precision of speed measurement data.

**Figure 6:**
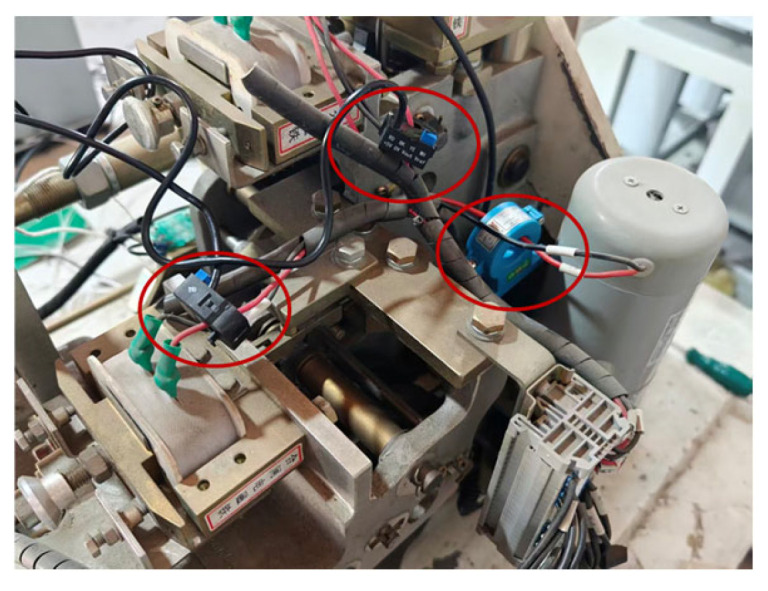
Opening/closing current and energy storage motor current sensors.

For the electrical state perception of the GIS circuit breaker operating mechanism, this system uniformly selects Hall effect sensors as core components to achieve non-invasive monitoring of the opening/closing coils and the energy storage motor loop, as shown in Figure 6. During the system operation phase, by synchronously collecting and analyzing the real-time current waveform characteristics of the coils and motor, it is possible not only to quantitatively evaluate the operating performance of electrical components but also to utilize the coupling relationship between current and mechanical load to indirectly invert the mechanical health status of the spring mechanism.

### 2.2. Fault Simulation Experiments

**Figure 7:**
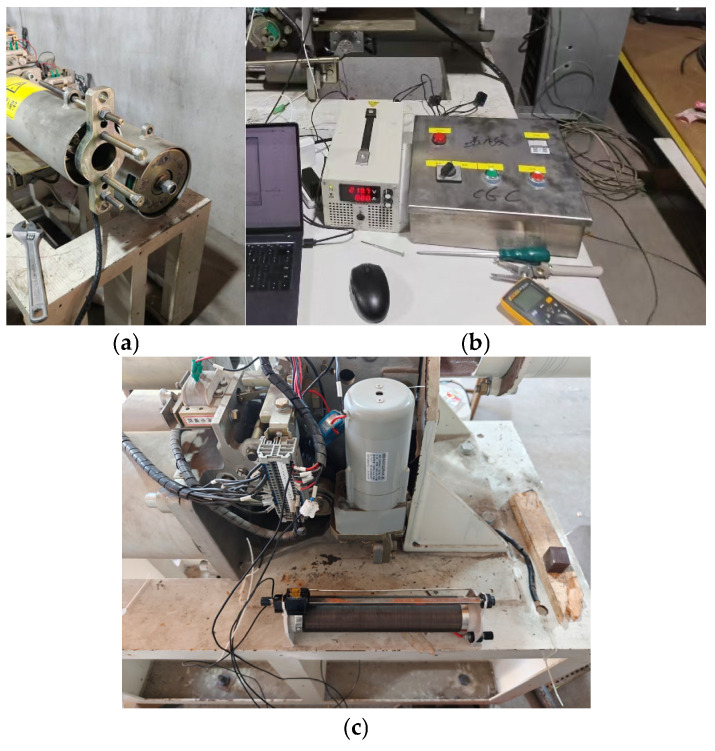
Fault simulation experiments: (**a**) opening/closing spring fatigue; (**b**) opening/closing coil voltage deviation; (**c**) energy storage motor coil aging.

This paper artificially sets up simulations for faults under various actual working conditions, including opening spring fatigue, closing spring fatigue, opening/closing coil voltage deviation, and energy storage motor coil aging. The simulation methods for each fault are shown in Figure 7.

Since the number of circuit breaker operations in actual engineering is very limited, excessive operations may have a certain impact on the circuit breaker, leading to deviations in measured sensor data. Therefore, this paper obtains small-sample data through experiments, using manual triggering of the mechanism box to obtain data.

**Normal Condition**: We ensure that the mechanism is at factory settings and perform operations without changing the initial state of the mechanism to obtain 100 sets of data under normal conditions.

**Opening/Closing Spring Fatigue Fault**: We loosen the opening spring bolt by 1 mm. A total of 100 sets of fault experimental data were obtained. According to Hooke’s law, a 1 mm reduction in pre-compression length translates to a 3–5% drop in the nominal static holding force, which closely corresponds to the actual natural stress relaxation and cumulative fatigue observed in circuit breakers after 5 to 8 years of field operation. After the experiment, a spring tool was installed to press the spring back to the initial position, ensuring spring reset through pressure sensor readings. The closing spring bolt was loosened in the same way to obtain 100 sets of experimental data.

**Opening/Closing Coil Voltage Deviation**: An external DC power supply box was used to directly adjust the power supply amplitude of the opening/closing coils. The initial power supply voltage was 220 V, and the fault voltage was set to 198 V. A total of 100 sets of fault experimental data were obtained.

**Energy Storage Motor Coil Aging**: A 50-ohm sliding rheostat was connected in series in the energy storage motor coil loop to simulate the motor coil aging fault. The sliding rheostat was adjusted to insert a 5-ohm resistor for operation, and a total of 100 sets of experimental data were obtained.

During the data acquisition process, the sampling frequency for all multi-source sensors was uniformly set to 10 kHz to ensure a high resolution. The recording duration for each operation was set to 2000 ms, which is sufficient to fully capture both the transient opening/closing mechanical actions and the subsequent operation of the energy storage motor.

## 3. Data Processing

### 3.1. Sensor Measured Data

**Figure 8:**
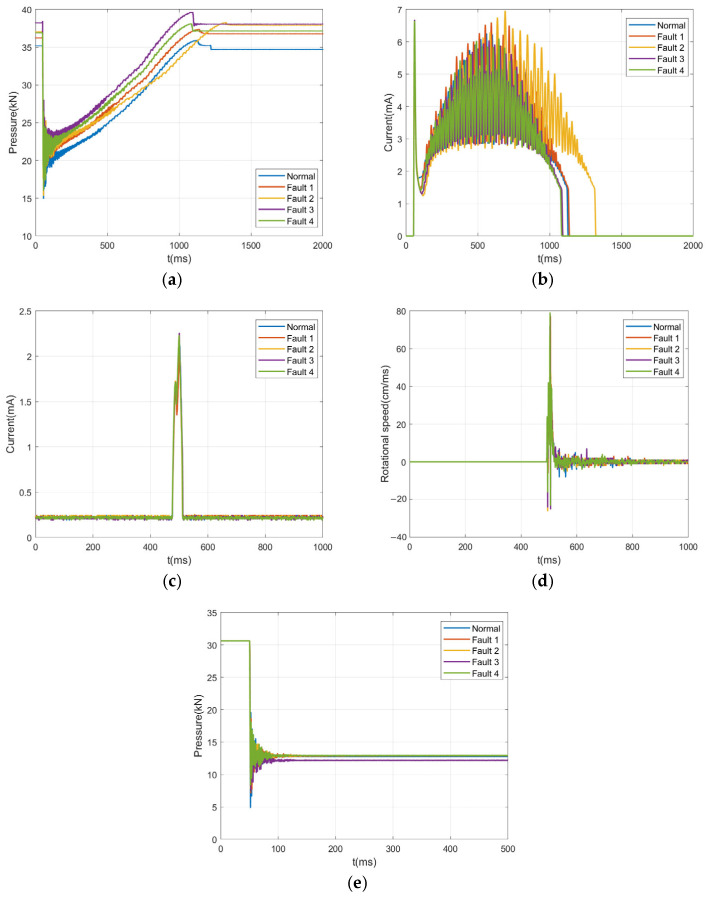
The measured data curves of sensors: (**a**) closing pressure; (**b**) energy storage motor current; (**c**) opening current; (**d**) opening speed; (**e**) opening pressure.

The measured sensor data obtained through experiments are shown in Figure 8, where Faults 1 to 4 correspond to opening spring fatigue, closing spring fatigue, opening/closing coil voltage deviation, and energy storage motor coil aging, respectively.

### 3.2. Sensor Signal Denoising

The on-site operating environment of high-voltage circuit breakers is complex, and the coil current and MEMS pressure signals collected by sensors are often mixed with high-frequency electromagnetic noise. These noises can mask key inflection points and extreme points of the signal. Although traditional low-pass filters (such as Butterworth) can remove noise, they easily cause smooth distortion of the signal waveform, leading to current peak attenuation or phase lag, which in turn affects the accuracy of subsequent feature extraction.

To effectively filter out high-frequency noise while maximizing the preservation of the original geometric features of the signal (such as peak height, waveform width, and edge steepness), this paper selects the Savitzky–Golay (S-G) digital filter to preprocess the original multi-source signals. The core physical features extracted from the waveforms, such as the peak coil current ($i_{c}$) and the maximum travel speed ($v_{m}$), are highly transient and rely on absolute geometric extremes. The optimality of the S-G filter lies in its local polynomial least-squares fitting mechanism, which inherently preserves the higher-order moments of the signal. This property allows the filter to effectively smooth out high-frequency random electromagnetic noise while strictly maintaining the true amplitude of sharp peaks and the steepness of transient edges. By preventing peak attenuation and phase distortion, the S-G filter guarantees the high fidelity of the extracted physical features, making it the optimal choice for the proposed diagnostic framework.

The core principle of S-G filtering involves selecting a sliding window of length 2m+1 in the time domain and performing least-squares fitting on the discrete data points within the window using a p-th-order polynomial [30].

Let the original discrete signal sequence be denoted as $x\left[n\right]$. For any arbitrary time instant n, where the sliding window interval centered at n is defined as $\left[n-m,n+m\right]$, the 2m+1 data points within the window can be represented as a vector $x_{win}$. Assuming that the degree of the fitting polynomial $f\left(i\right)$ is p (where p<2m+1), its mathematical expression is given by:

(1) $$f\left(i\right)=\sum_{k=0}^{p}b_{k}i^{k},\;i\in \{-m,-m+1,\ldots ,0,\ldots ,m\}$$

where i represents the relative coordinate position within the window and $b_{k}$ denotes the polynomial coefficients to be solved.

The mathematical essence of S-G filtering is to solve for a set of optimal coefficients $b={\left[b_{0},b_{1},\ldots ,b_{p}\right]}^{T}$ such that the Mean Squared Error (MSE) between the fitting polynomial and the original data points is minimized. The objective function J is constructed as follows:

(2) $$J=\sum_{i=-m}^{m}{\left(f\left(i\right)-x\left[n+i\right]\right)}^{2}=\sum_{i=-m}^{m}{\left(\sum_{k=0}^{p}b_{k}i^{k}-x\left[n+i\right]\right)}^{2}$$

To solve this least-squares problem, it is reformulated into matrix form. We define the observation vector y and the design matrix (Vandermonde matrix) S as

(3) $$y={\left[\begin{array}{c} x\left[n-m\right]\\ x\left[n-m+1\right]\\ \vdots \\ x\left[n+m\right] \end{array}\right]}_{\left(2m+1\right)\times 1},\;S={\left[\begin{array}{cccc} {\left(-m\right)}^{0} & {\left(-m\right)}^{1} & \ldots & {\left(-m\right)}^{p}\\ {\left(-m+1\right)}^{0} & {\left(-m+1\right)}^{1} & \ldots & {\left(-m+1\right)}^{p}\\ \vdots & \vdots & \ddots & \vdots \\ m^{0} & m^{1} & \ldots & m^{p} \end{array}\right]}_{\left(2m+1\right)\times \left(p+1\right)}$$

Consequently, the fitting model can be expressed as a system of linear equations:

(4) y=S⋅b+ε

where ε is the residual vector. According to the least-squares principle, setting the partial derivative of the objective function with respect to the coefficient vector b to zero (i.e., $\frac{\partial J}{\partial b}=0$) yields the analytic solution for the optimal estimate $\hat{b}$:

(5) $$\hat{b}={\left(S^{T}S\right)}^{-1}S^{T}y$$

The filtered output $\hat{x}\left[n\right]$ corresponds to the polynomial-fitted value at the window center position (i.e., relative coordinate: i=0). According to the polynomial definition, when i=0, $f\left(0\right)=b_{0}$. Therefore, the filtering output depends solely on the first element $b_{0}$ of the coefficient vector $\hat{b}$.

Let the projection matrix be $H={\left(S^{T}S\right)}^{-1}S^{T}$. The filtering process can then be simplified as a convolution operation between the original signal and a specific convolution kernel:

(6) $$\hat{x}\left[n\right]={\hat{b}}_{0}=\sum_{j=-m}^{m}h_{0,j}\cdot x\left[n+j\right]$$

where $h_{0,j}$ represents the weighting coefficients corresponding to the first row of matrix H.

Based on experimental analysis, this paper selects a window half-width of m=25 (i.e., a window length of N=51) and a polynomial order of p=3. Compared to traditional moving average filtering (which corresponds to the special case where p=0), the third-order S-G filter utilizes high-order moment information to smooth noise while effectively preserving high-frequency edge features.

### 3.3. Sensor Curve Feature Information Extraction

**Figure 9:**
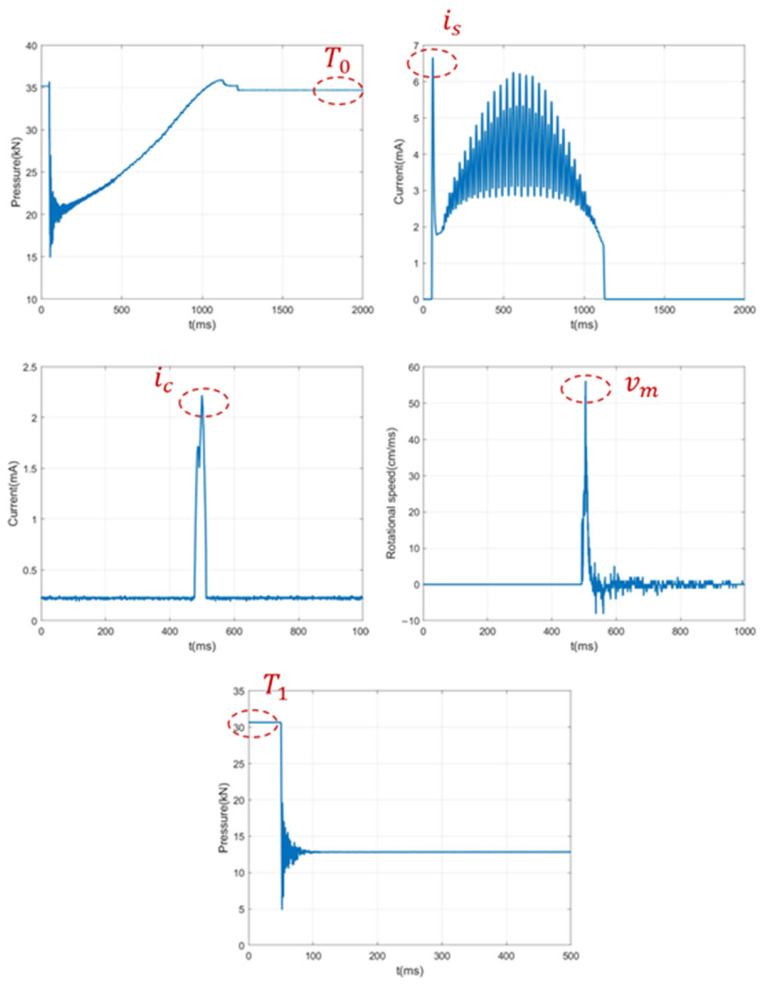
Feature value extraction.

The characteristic values in sensor curves can accurately reflect the operating status of the spring operating mechanism; here, effective characteristic quantities of each curve are extracted. For the closing and opening spring pressure curves, the static pressure values $T_{0}$ and $T_{1}$ during spring compression are obtained, representing the force under the spring’s compressed state, i.e., reflecting the fatigue degree of the spring. $i_{s}$ represents the maximum motor starting current, which can be used to judge changes in the energy storage motor coil resistance. $i_{c}$ represents the maximum current of the opening/closing coil, which can fully reflect the motion characteristics during the opening process. $v_{m}$ represents the maximum opening speed, which can reflect the status of key components such as the circuit breaker energy storage spring, the buffer spring, and moving contacts (Figure 9).

## 4. Diagnosis Algorithm Based on Stacking Ensemble Learning

When addressing the characteristics of scarce fault samples and complex multi-source feature space distribution in high-voltage circuit breakers, it is often difficult to simultaneously account for fitting accuracy on training data and generalization performance on unknown data using a single classifier. To effectively solve the Bias–Variance Tradeoff problem under small-sample conditions, this paper constructs a two-layer Stacking ensemble learning model combined with K-fold cross-validation. This model aims to map the original physical feature space to a high-dimensional decision probability space by integrating base classifiers with different mathematical mechanisms and uses a meta-learner to achieve adaptive fusion and error correction of multi-view fault features [31].

### 4.1. Selection of Heterogeneous Base Classifiers

To build an ensemble model with strong generalization capabilities, the level-0 base learners must strictly satisfy the “diversity” principle. The rationale for selecting the Support Vector Machine (SVM), the Random Forest (RF), and K-Nearest Neighbors (KNNs) specifically lies in their strong mathematical and geometric complementarity within the high-dimensional feature space.

Geometrically, the SVM constructs global nonlinear decision boundaries by mapping features into a higher-dimensional space via the RBF kernel, effectively isolating heavily overlapping classes. However, global optimization may obscure local micro-structures. KNNs perfectly complement this by focusing solely on local manifold structures based on the Euclidean distance, which is crucial for identifying ‘familial aggregation’ defects characterized by high local density in the feature space. Meanwhile, unlike the distance-metric dependence of the SVM and KNNs, the RF partitions the feature space orthogonally based on information entropy. This tree-based mechanism is naturally immune to distance metric distortion caused by unscaled noise and can inherently suppress redundant features. Through this combination—the global boundary (SVM), the local neighborhood (KNN), and orthogonal feature splitting (RF)—the multi-source features are comprehensively parsed without algorithmic homogenization.

For the level-1 meta-learner, Logistic Regression (LR) is selected to prevent the severe overfitting that would occur if a complex nonlinear model was applied to a limited sample size. LR acts as an adaptive, linear weighted voting mechanism, evaluating the confidence of each base learner’s probabilistic output and seamlessly fusing their complementary advantages. First, the Support Vector Machine (SVM) is selected as a base model capable of capturing global nonlinear boundaries. The SVM is a discriminative model based on the Structural Risk Minimization (SRM) principle in statistical learning theory. Compared with traditional algorithms pursuing Empirical Risk Minimization, the SVM aims to find an optimal hyperplane satisfying the maximum classification margin, giving it significant advantages when dealing with the “small sample, high dimension” classification problems faced in this paper. To address the linearly inseparable nature of circuit breaker features, the Radial Basis Function (RBF) is introduced as the kernel function, and its optimization objective is to solve a convex quadratic programming problem with slack variables ξ:

(7) $$\begin{array}{ll} \underset{w,b,\xi }{min}\; & \frac{1}{2}{\left\|w\right\|}^{2}+C\sum_{i=1}^{N}\xi_{i}\\ s.t.\; & y_{i}\left(w^{T}\phi \left(x_{i}\right)+b\right)\geq 1-\xi_{i},\;\xi_{i}\geq 0 \end{array}$$

Physically, the SVM excels at capturing nonlinear global boundaries between different fault categories, as it is capable of establishing a stable classification plane through support vectors even with very few training samples.

Second, a Random Forest (RF) is introduced to enhance the model’s robustness to sensor noise. The RF is an algorithm based on the Bagging ensemble strategy, making joint decisions by constructing multiple decorrelated decision trees. Its core mechanism lies in “double randomness”: generating differentiated training sets via Bootstrap resampling and randomly selecting feature subspaces based on the Gini Index minimization criterion during node splitting. The final fault category determination is decided by the voting results of all decision trees:

(8) $$H\left(x\right)=arg\underset{y}{max}\sum_{t=1}^{T}I\left(h_{t}\left(x\right)=y\right)$$

The RF can automatically screen out key variables sensitive to faults from high-dimensional features, reducing the interference of invalid features.

Finally, K-Nearest Neighbors (KNNs) are utilized to mine the local manifold structure of the feature space. The KNN method is a non-parametric lazy learning algorithm that does not pre-assume the global distribution form of data but directly measures local similarity based on the Euclidean distance in the feature space. For a sample to be diagnosed, the algorithm makes decisions through the following weighted neighborhood voting mechanism:

(9) $$y=arg\underset{c}{max}\sum_{x_{i}\in N_{K}(x)}\frac{1}{{\left\|x-x_{i}\right\|}^{2}}\cdot I\left(y_{i}=c\right)$$

The mechanical performance of high-voltage circuit breakers often possesses “familial aggregation” (i.e., equipment of the same batch or aging degree shows similar characteristics). When the global features of a test sample are not obvious but highly similar to specific fault samples in the training set, the KNN algorithm can provide a key local diagnostic basis to supplement global models.

### 4.2. Meta-Feature Generation Based on 5-Fold Cross-Validation

In constructing the second-layer input, directly inputting the training data predictions into the meta-learner would lead to severe Data Leakage and overfitting. Therefore, this chapter adopts a strict 5-fold cross-validation strategy to generate Meta-Features. The specific process is as follows: randomly divide the original training set into five mutually exclusive subsets. For each base classifier, sequentially select one subset as the validation set and the remaining four subsets as the training set for model training and inference. Through circular traversal, the Out-of-Fold Prediction probability vector for the entire training set is obtained. Simultaneously, the base models are retrained using the full training set to predict the test set. Finally, the posterior probability vectors output by the SVM, the RF, and KNNs are concatenated to form the meta-feature matrix (Z) containing multi-model decision perspectives, serving as input for the second-layer model:

(10) $$Z=\left[P^{\left(SVM\right)},P^{\left(RF\right)},P^{\left(KNN\right)}\right]$$

This strategy ensures that the training data for the meta-learner is “unseen” by the base models, thereby truly reflecting the base models’ generalization ability on unseen samples.

### 4.3. Meta-Learner Design and Adaptive Fusion

In the design of the second-layer meta-learner (level-1 meta-learner), the Logistic Regression (LR) model, which has a simple structure and strong interpretability, is selected to perform secondary fusion of the base models’ prediction results. The LR model maps the input meta-feature vector **z** to the final fault probability via the Sigmoid function

(11) $$P\left(y=c|z\right)=\frac{1}{1+exp\left(-\left(\omega^{T}z+b\right)\right)}$$

and solves for the optimal weight configuration of each base model by minimizing the logarithmic loss function $J\left(\omega ,b\right)$:

(12) $$J\left(\omega ,b\right)=-\frac{1}{N}\sum_{i=1}^{N}\sum_{c=1}^{C}I\left(y_{i}=c\right)logP\left(y_{i}=c|z_{i}\right)$$

This mechanism effectively endows the model with “adaptive weighting” capability: if a base model has high prediction confidence and accuracy for a specific fault category, LR will automatically assign a larger weight to its corresponding probability component and conversely reduce its influence. Through this hierarchical architecture, the Stacking model not only integrates the physical features of MEMS sensors but also effectively avoids the risk of overfitting under small samples through the cross-validation mechanism, achieving high-precision fault diagnosis.

### 4.4. Experimental Results and Data Analysis

To systematically verify the effectiveness and superiority of the multi-source fault diagnosis method based on Stacking ensemble learning proposed in this paper, an experimental dataset containing five typical working conditions—the normal state, opening spring fatigue, closing spring fatigue, opening/closing coil voltage deviation, and motor coil aging—was constructed based on a 220 kV high-voltage circuit breaker true-type experimental platform, with a total sample size of 500 sets. In the model training and evaluation stage, to test the model’s generalization ability under unknown conditions more rigorously, the dataset was divided into a training set and a testing set by random sampling, with the training set accounting for 40% (200 sets) and the testing set accounting for 60% (300 sets). All algorithm models were built and run on the MATLAB R2024b platform.

Prior to evaluating the models on the testing set, the hyperparameters of the base classifiers (SVM, RF, and KNN) were rigorously tuned to ensure optimal performance and prevent overfitting. Rather than relying on empirical selection, a Grid Search with a 5-fold cross-validation optimization procedure was employed using the training data. This method exhaustively searches through a predefined hyperparameter grid to find the combination that yields the highest average cross-validation accuracy.

Specifically, for the SVM model with an RBF kernel, the penalty parameter C and kernel coefficient γ were optimized, resulting in final values of C=10 and γ=0.1. For the Random Forest (RF) model, to balance model complexity and generalization, the optimal number of trees and the maximum depth were determined to be n_estimators=100 and max_depth=8. For the KNN model, the optimal number of neighbors was found to be K=5 when utilizing the Euclidean distance as the metric. These optimized base models were subsequently integrated into the Stacking framework.

**Figure 10:**
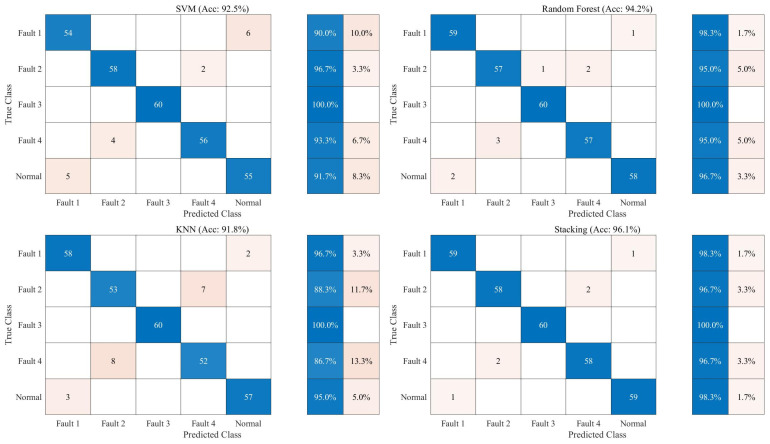
Confusion matrix comparison.

Figure 10 shows the comparison results of confusion matrices for the three base classifiers (SVM, Random Forest (RF), and KNN) and the Stacking ensemble model proposed in this paper on the test set. Faults 1 to 4 correspond to opening spring fatigue, closing spring fatigue, opening/closing coil voltage deviation, and energy storage motor coil aging, respectively.

Figure 10 displays the comparison of confusion matrices for the four algorithm models on the test set, intuitively revealing the specific misjudgment distribution of different models in multi-classification tasks. The SVM model shows significant confusion when handling “Normal” and “Fault 1” states, with about 8.3% of samples being misclassified. This indicates that the fault features corresponding to “Fault 1” highly overlap with the normal state in the hyperplane mapping space, and it is difficult to achieve complete decoupling using a single linear or kernel function mapping. Similarly, the KNN model has limitations in distinguishing “Fault 2” and “Fault 4”, with misjudged samples mainly concentrated between these two types of faults, reflecting that the metric based on the Euclidean distance lacks robustness when dealing with these two faults with similar local features.

To provide a comprehensive evaluation for multi-class fault diagnosis, the Precision, Recall, and F1-score for each class were calculated (Table 1). The macro-average F1-score reached 98.00%, which proves that the Stacking model maintains a highly balanced performance across all fault categories without suffering from class-biased predictions.

In sharp contrast, the Stacking ensemble model significantly corrects the specific misjudgments of the aforementioned base models. By introducing a meta-learner to perform secondary learning on the prediction probabilities of base models, the Stacking model successfully utilizes the Random Forest’s advantage in feature selection to compensate for the SVM’s deficiency in boundary division. As can be seen from the figure, the Stacking model has a more obvious dominant advantage on the diagonal, not only minimizing the number of misjudgments between “Normal” and “Fault 1” but also effectively improving the recognition accuracy of complex faults such as “Fault 3”, proving that the ensemble strategy possesses stronger error correction capability and improved robustness on unseen samples from the studied configuration when processing heterogeneous fault features.

**Figure 11:**
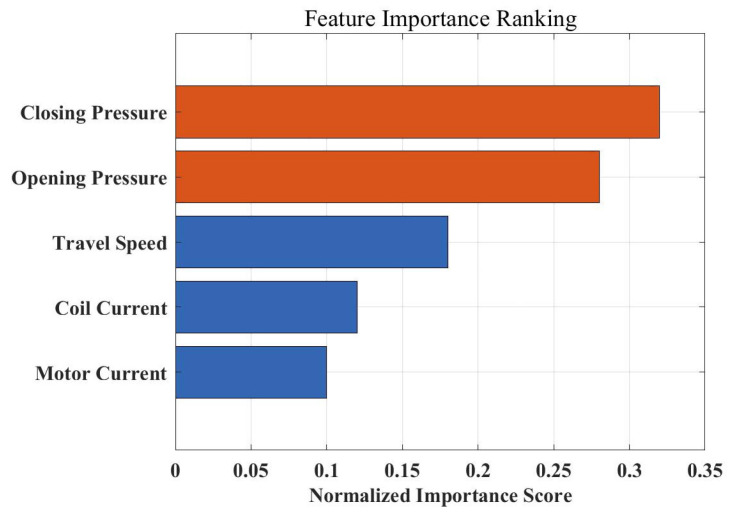
Feature importance ranking.

To explore the contribution of different physical quantities to circuit breaker status assessments, this paper quantified and ranked the importance of input features based on the Out-of-Bag (OOB) error analysis technique of the Random Forest algorithm, as shown in Figure 11. The experimental results show that feature importance presents a significant physical hierarchy: “Closing Pressure” and “Opening Pressure” rank at the top in normalized importance scores, at 0.32 and 0.28 respectively. This result aligns highly with the physical mechanism of the circuit breaker operating mechanism, indicating that pressure signals collected by MEMS sensors can directly reflect subtle changes in spring potential energy and mechanism resistance, serving as core criteria for distinguishing different mechanical faults. Following closely is “Travel Speed,” which, as direct kinematic feedback of mechanism action, has an important reference value for judging jamming-type faults. In comparison, the importance of “Coil Current” and “Motor Current” is relatively lower, indicating that although electrical quantities can reflect the state of the control loop, mechanical and kinematic features have higher sensitivity and discrimination in refined diagnosis targeting mechanical characteristics.

Furthermore, to address potential concerns regarding the stability of the results under the unconventional 40/60 split, a full 5-fold cross-validation step was conducted on the entire dataset. The Stacking model achieved an average diagnostic accuracy of 97.2% across the five folds. This full cross-validation confirms that the model’s exceptional performance is highly robust and not overly reliant on any specific random data partition, thereby demonstrating its strong generalization capability under limited-sample conditions.

**Figure 12:**
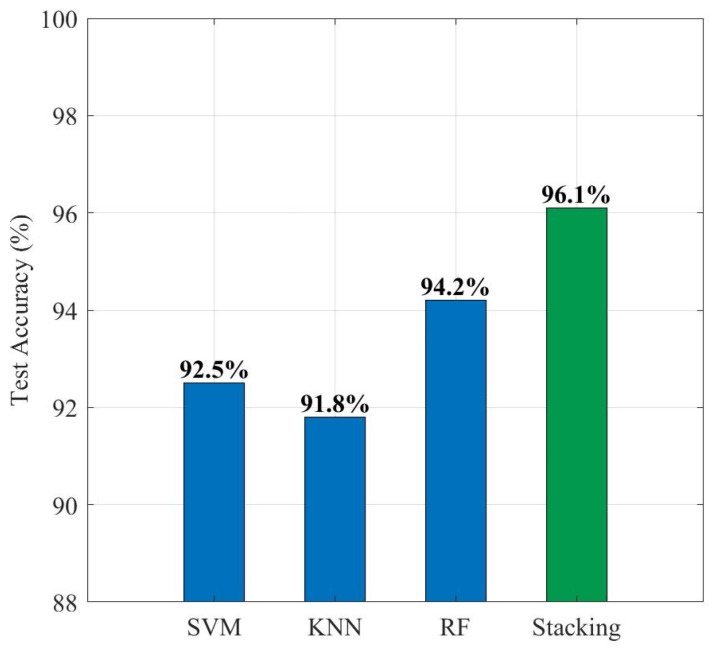
Performance comparison of different algorithms.

Figure 12 provides a quantitative comparison of the overall accuracy of different diagnostic algorithms on the test set. It is clearly visible from the data that the performance of single base classifiers is limited by their respective algorithmic biases: the accuracy of the KNN and SVM are 91.8% and 92.5%, respectively, making it difficult to break the 93% bottleneck; the Random Forest algorithm, by virtue of its strong anti-noise ability, achieved a relatively high accuracy of 94.2% but still had a small number of misjudgments. In comparison, the Stacking ensemble model proposed in this paper achieved the highest accuracy of 96.1%. Compared to the best-performing base model (RF), the Stacking strategy achieved a performance improvement of 1.9 percentage points; compared to the weakest base model (KNN), the improvement amplitude reached 4.3%. This result fully verifies that the Stacking framework can effectively integrate the decision boundaries of different base learners, reduce the variance and bias risks of single models, and thus achieve precise diagnosis of multiple types of faults in high-voltage circuit breakers while ensuring high reliability.

## 5. Conclusions

This paper carried out research on a diagnostic method based on multi-source sensing and Stacking ensemble learning, addressing the difficulties of single features and scarce samples in the fault diagnosis of high-voltage circuit breaker spring operating mechanisms. Through theoretical analysis, system construction, and true-type experimental verification, the main conclusions determined are as follows:

(1) The introduction of MEMS pressure features significantly improved the diagnostic dimension. Experimental results show that the closing and opening holding pressures collected by MEMS sensors rank at the forefront of feature importance sorting (normalized scores of 0.32 and 0.28 respectively). This confirms that static pressure features are core criteria characterizing spring fatigue and mechanism health status, effectively making up for the deficiency of traditional current and travel monitoring in static perception.
(2) The Stacking ensemble strategy effectively overcame the risk of small-sample overfitting. In the diagnostic experiment of 300 sets of test samples, the comprehensive accuracy of the Stacking model reached 96.1%. Compared to the single SVM (92.5%), KNN (91.8%), and Random Forest (94.2%) algorithms, the Stacking model significantly corrected misjudgments between specific faults (such as spring fatigue and the normal state) by integrating the decision boundaries of heterogeneous base classifiers, demonstrating enhanced reliability and robustness on the specific circuit breaker under study.
(3) Multi-source feature fusion achieved precise decoupling of single-point faults. By fusing multi-dimensional information such as electrical (current), motion (travel), and mechanical (pressure) data, the method proposed in this paper can effectively identify various typical single-point defects including spring fatigue, mechanism jamming, and coil faults. Specifically, for single-fault categories that exhibit high feature similarity in a single sensing domain, the multi-source information complementary mechanism drastically reduced the false alarm rate.

In summary, the method proposed in this paper realizes a complete closed loop from data acquisition to intelligent diagnosis without damaging the original structure of the equipment, providing a reliable technical path for the transformation of high-voltage circuit breakers from “periodic maintenance” to “condition-based maintenance”.

However, it is important to explicitly note the boundaries of the current study. The experimental validations and conclusions drawn herein are currently limited to the specific 220 kV circuit breaker configuration, and the diagnostic framework focuses exclusively on single-point faults. The analysis of borderline cases, such as the exact separation of overlapping or compound defects occurring simultaneously, has not been validated. Therefore, future work will further study the transfer learning ability of this model on different mechanism models and investigate advanced decoupling algorithms for simultaneous compound faults to enhance its universality in ubiquitous power Internet of Things scenarios.

## Figures and Tables

**Table 1:** Classification of common faults of spring-operated mechanism-based circuit breakers.

Class	Precision (%)	Recall (%)	F1-Score (%)
Normal	98.33	98.33	98.33
Fault 1	98.33	98.33	98.33
Fault 2	96.67	96.67	96.67
Fault 3	100.00	100.00	100.00
Fault 4	96.67	96.67	96.67
Macro-Average	98.00	98.00	98.00

## Data Availability

The data presented in this study are available on request from the first author.
